# Targeted lipidomics analysis of possible molecular mechanisms of lipid changes in temporal lobe epilepsy models

**DOI:** 10.3389/fphar.2024.1531524

**Published:** 2025-01-09

**Authors:** Huaiyu Sun, Xuewei Li, Zhiqing Chen, Hongmei Meng

**Affiliations:** ^1^ Department of Neurology, The First Hospital of Jilin University, Changchun, Jilin, China; ^2^ Department of Radiology, The First Hospital of Jilin University, Changchun, Jilin, China

**Keywords:** targeted lipidomics analysis, epilepsy, temporal lobe epilepsy, molecular mechanism, therapeutic target

## Abstract

**Background:**

Lipids are vital biomolecules involved in the formation of various biofilms. Seizures can cause changes in lipid metabolism in the brain. In-depth studies at multiple levels are urgently needed to elucidate lipid composition, distribution, and metabolic pathways in the brain after seizure.

**Methods:**

In this research, a cutting-edge targeted quantitative lipidomics study was conducted on the hippocampal tissues of six rats with temporal lobe epilepsy and six normal rats. Accurate lipid quantification based on linear equations was calculated using an internal standard. The lipids were quantitatively and qualitatively analyzed by ultra-high performance liquid chromatography (UPLC) and mass spectrometry (MS).

**Results:**

A total of 21 lipid classes were identified. Among them, the most abundant were triacylglycerol (TG), phosphatidyl ethanolamine (PE-P), and fatty acids (FA). Cholesteryl ester (ChE) exhibits the most considerable difference between the normal and epileptic samples. ChE was found to be the most significantly upregulated lipid, while FA was observed to be the most significantly downregulated lipid.

**Conclusion:**

Based on the absolute quantitative analysis of lipids in rat hippocampal specimens, the contents and change trends of different lipids were observed. Upregulation of ChE and dihydroceramide (DHCer) was observed, and an analysis of the distribution changes elucidated the causes and possible molecular mechanisms of lipid accumulation in temporal lobe epilepsy. The results and methods described provide a comprehensive analysis of lipid metabolism in temporal lobe epilepsy and a new therapeutic target for the treatment of epilepsy.

## 1 Introduction

Epilepsy is one of the most common neurological disorders affecting more than 50 million people. Temporal lobe epilepsy (TLE) is the most common focal epilepsy in adults (Engel and International League Against Epilepsy ILAE, 2001). It is characterized by unprovoked recurrent focal seizures in the temporal lobe, resulting in a wide range of clinical manifestations (Cavarsan et al., 2018). Approximately 30% of patients eventually progress to drug-resistant epilepsy with unpredictable recurrent seizures, for which treatment options are limited, besides surgery (Löscher et al., 2020; Thijs et al., 2019). Hippocampal morphological changes such as neuronal death, gliosis, dendritic alterations, and mossy fiber sprouting are the major pathohistological features of drug-resistant epilepsy (Verkhratsky and Nedergaard, 2018; Vezzani et al., 2019).

Lipids are important biomolecules involved in the building up of various biological membranes and represent 60% of the human brain content (Dawson, 2015). By analyzing the magnetic resonance spectroscopy data from TLE patients, Yan et al. found that lipid signaling was significantly increased in TLE participants’ epileptogenic focus compared to the contralateral normal side of the same participant, indicating abnormal lipid accumulation in the epileptic brain. They then used lipid-detecting antibodies to label cortical samples from TLE participants and “normal” controls (participants undergoing surgery for benign brain tumors or trauma surgery), and the results indicated that TLE patients had significantly increased lipid droplet levels in neurons and astrocytes in the cortex (Chen et al., 2023). Previous studies have shown that certain anti-seizure medications (ASMs) adversely impact lipid concentrations in people with epilepsy. When we analyzed the impact of individual ASMs used by more than 15% of the cohort on the lipid levels, participants using valproic acid (VPA) showed lower high-density lipoprotein and higher triglyceride levels compared to participants not using VPA (Pylvänen et al., 2003). These findings suggest that lipid metabolism plays an important role in epilepsy.

Therefore, understanding lipid changes after seizures may lead to a better understanding of lipid metabolic alterations, which could provide clues for further control of neurons affected by lipid changes. Although lipidomics has been widely used in recent years and the role of lipid decomposition and oxidation has been extensively studied, lipid metabolism in epilepsy, especially in hippocampal neurons in temporal lobe epilepsy, has been poorly studied. Therefore, the purpose of this study is to use targeted lipidomics to quantitatively record the changes in various lipid levels after epileptic seizure and provide a new idea for the study of the treatment of epilepsy through lipid metabolism.

## 2 Materials and methods

### 2.1 Animals and chemicals

A total of 12 healthy adult male Wistar rats (280–300 g) were purchased from Beijing Weitong Lihua Experimental Animal Technology Co., Ltd. The animals were uniformly raised in the animal room of the Department of Neurology, Translational Medical College, Jilin University, with the temperature maintained at 23 ± 3°C and provided free access to food and water. Kainic acid (KA) was purchased from Sigma-Aldrich (Shanghai Trading Co., Ltd.).

A total of six rats were assigned to the healthy control group. The other six rats were used to construct the models of status epilepticus (SE) by injecting KA into the amygdala. A total of six rats were successfully constructed as SE models. Epilepsy group rats were sacrificed at 24 h after the amygdala-kindling surgery, and the healthy control group rats were killed to collect the hippocampus tissues.

### 2.2 KA-induced epileptic rat model

The rats were anesthetized with inhalational isoflurane gas (3% for induction and then 1.5% for maintenance). The head of the rat was fixed on the stereotaxic instrument with the horizontal scales of the two external auditory canals maintained at the same level. The incisor brackets were adjusted so that the anterior and posterior fontanelles of the rat were parallel to the desktop, that is, the head of the rat was kept horizontal. Then, the skin of the head of the rat was prepared and disinfected, and a longitudinal incision 2–3 cm long was made along the middle of the head to separate the skin, fascia, and other soft tissues to expose the coronal suture as well as the anterior and posterior fontanelles. An electric cranial drill for small animals was used to drill through the skull, and it was positioned at 2.5 mm behind the bregma and 4.5 mm on the right side of the surface of the dura mater. Then, the microinjector was fixed, with the needle slowly inserted to the depth of 8.5 mm from the skull, and 0.6 μg KA (in 0.6 μL of 1 μg/μL KA solution) was slowly injected. The needle was maintained inside for 10 min after the injection and then slowly withdrawn. The skin incision was aseptically sutured, with the rat ear tag used to mark the animals that had undergone surgery. The rats were kept warm after the surgery until they were fully awake. The successful construction of the rat model of SE was evaluated according to the Racine scales that is, grade 0: no behavioral changes; grade 1: facial clonus, including blinking, whiskers, and rhythmic chewing; grade II: grade I behaviors plus rhythmic nodding or tail flicking; grade III: grade II behaviors with myoclonus of the forelimbs but no hindlimb upright position; grade IV: grade III behaviors as well as hindlimb erection; and grade V: generalized tonic–clonic seizures and loss of postural control with fallings. The successful establishment of the rat model of SE was determined based on the occurrence of seizures categorized as grade IV or above within 1 h after the surgery. These seizures of grade IV and above were terminated by intraperitoneal injection of diazepam (8 mg/kg).

#### 2.2.1 Sample pretreatment method

Lipids were extracted according to the MTBE method. Briefly, samples were first mixed with 200 µL methanol, and then 10 µL internal lipid standards and 800 µL MTBE were added. The mixture was adequately vortexed, sonicated for 20 min at 4°C, and then kept for 30 min at room temperature. After that, 200 µL of MS-grade water was added, and the mixture was vortexed and centrifuged at 14,000 rpm for 15 min at 4°C. The upper organic solvent layer was obtained and dried under nitrogen. For LC–MS analysis, the samples were re-dissolved in 200 µL of IPA/ACN (9:1, v/v) solvent and centrifuged at 14,000 rpm at 4°C for 15 min, and then the supernatant was injected.

### 2.3 LC–MS/MS method for lipid analysis

The analysis was performed on a UHPLC system (LC-30AD, Shimadzu) coupled with QTRAP MS (6500+, Sciex). The analytes were separated on HILIC (Phenomenex, Luna NH2, 2.0 mm × 100 mm, 3 µm) and C18 column (Phenomenex, Kinetex C18, 2.1 × 100 mm, 2.6 μm). For C18 separation, the column temperature was set at 45°C. Mobile phase A: 70% acetonitrile + 30% H_2_O + 5 mM ammonium acetate; mobile phase B: IPA solution. A gradient (20% B at 0 min, 60% B at 5 min, 100% B at 13 min, and 20% B at 13.1–17 min) was then initiated at a flow rate of 0.35 mL/min. The sample was placed at 10°C during the whole analysis process. For amino separation, the column temperature was set at 40°C. Mobile phase A: 2 mM ammonium acetate +50% methanol + 50% acetonitrile; mobile phase B: 2 mM ammonium acetate +50% acetonitrile +50% water. A gradient (3% B at 0–3 min, from 3% to 100% B at 3–13 min, 100% B at 13–17 min, and 3% B at 17.1–22 min) was then initiated at a flow rate of 400 μL/min. 6500+QTRAP (AB SCIEX) was performed in the positive and negative switch modes. The ESI positive source conditions were as follows: source temperature: 400°C; ion source gas 1 (GS1): 50; ion source gas 2 (GS2): 55; curtain gas (CUR): 35; ion spray voltage (IS): +3,000 V. The ESI negative source conditions were as follows: source temperature: 400°C; ion source gas 1 (GS1): 50; ion source gas 2 (GS2): 55; curtain gas (CUR): 35; ion spray voltage (IS): -2,500 V. The MRM method was used for mass spectrometry quantitative data acquisition. The MRM ion pairs are shown in the attached file. The polled quality-control (QC) samples were set in the sample queue to evaluate the stability and repeatability of the system.

#### 2.3.1 Data processing

Sciex OS software was used to extract the peak of the original MRM data, the ratio of the peak area and the internal standard peak area of each substance was obtained, and then the content of each substance was further calculated. The extracted data were analyzed. The data analysis included identification quantity statistics, lipid composition analysis, and lipid difference analysis. Lipid composition analysis included lipid subclass composition and lipid content distribution analysis. Lipid difference analysis included lipid content, chain length, chain saturation analysis, and KEGG analysis. The analysis of lipid content changes involves the analysis of the whole, subclass, molecule, and other dimensions.

### 2.4 Statistical analysis

The data were explored by SIMCA (Version 16.0.2, Sartorius Stedim Data Analytics AB, Umea, Sweden) for multivariate statistical methods, including principal component analysis and partial least square discriminant analysis. The orthogonal partial least squares discriminant analysis (OPLS-DA) can filter out noise unrelated to classification information and improve the analytical ability and effectiveness of the model. The data were further analyzed using one-way ANOVA. The univariate analysis (*p*-value and fold changes) and multivariate analysis (variable importance in projection, VIP values) were performed. The screening criteria were as follows: *p* < 0.05, VIP >1, and FC > 1.5 or FC < 0.67. For visualization of the metabolic pathway analysis, MetaboAnalyst based on database sources including the Kyoto Encyclopedia of Genes and Genomes (KEGG) pathway database was used for the lipid pathway analysis. The SPSS software (version 20.0, SPSS Inc., Chicago, IL, United States) was used for the statistical analyses. Duncan’s multiple range test was used to compare the mean differences of TBARS, TVC, and lipid molecular species during refrigerated storage. A *p*-value <0.05 was determined to be a significant difference.

## 3 Results

### 3.1 Overall lipidomic analysis

**FIGURE 1:**
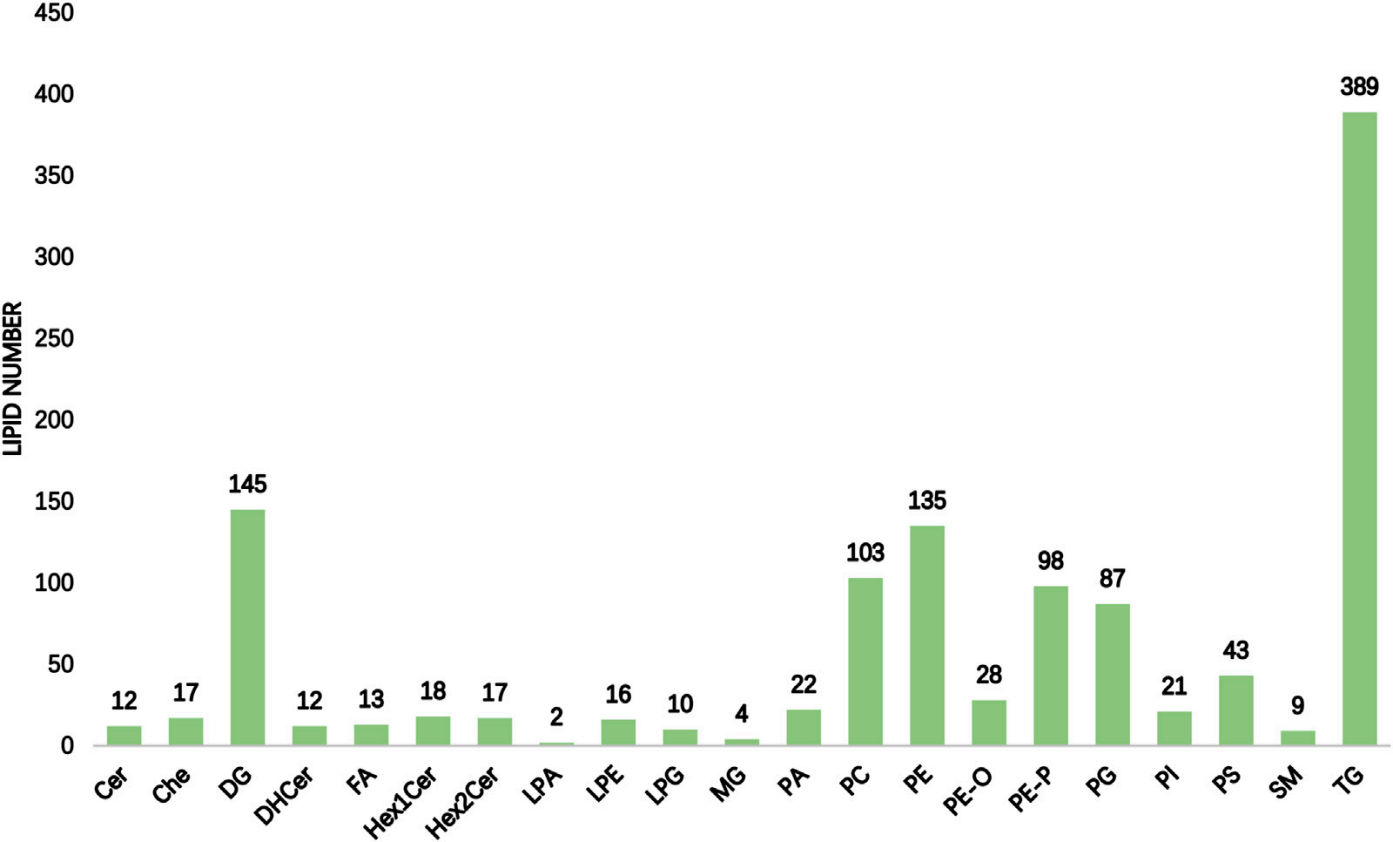
Statistical chart of lipid subclasses and lipid molecules (the horizontal coordinate of the figure indicates the detected lipid subclasses, and the vertical coordinate is the number of lipid molecules under the subclasses).

The International Lipid Classification and Nomenclature Committee divides lipid compounds into eight major categories. Each category can be further subdivided into different subclasses (lipid classes) based on the variations in their polar head groups. Within each subclass, further differentiation into specific molecular species (lipid species) is made based on factors such as carbon chain saturation or length. This forms a three-tier classification system of lipid compounds: category–subclass–molecular species. A total of 21 lipid classes and 1201 lipid species were detected and tentatively identified in the hippocampus of rats with TLE using UHPLC–MS/MS, as shown in Figure 1. In the hippocampus of epileptic rats, there were differences in six of 21 lipid classes, with increased levels of ChE and DHCer and decreased levels of FA, LPA, PE, and PE-O (Table 1).

**TABLE 1:** Impact of epileptic seizures on lipid metabolism in the rat hippocampus: quantitative analysis and differential expression of lipid classes.

Class	Mean epilepsy (ng/g)	Mean control (ng/g)	Ratio	*p-*value
ChE	433,758	210,369	2.061	<0.001
DHCer	28,644	24,722	1.158	0.039
FA	9,892,395	16,805,066	0.588	0.005
LPA	9,243	11,545	0.800	0.019
PE	1,559,435	2,316,263	0.673	0.020
PE-O	244,490	421,404	0.580	0.048

**FIGURE 2:**
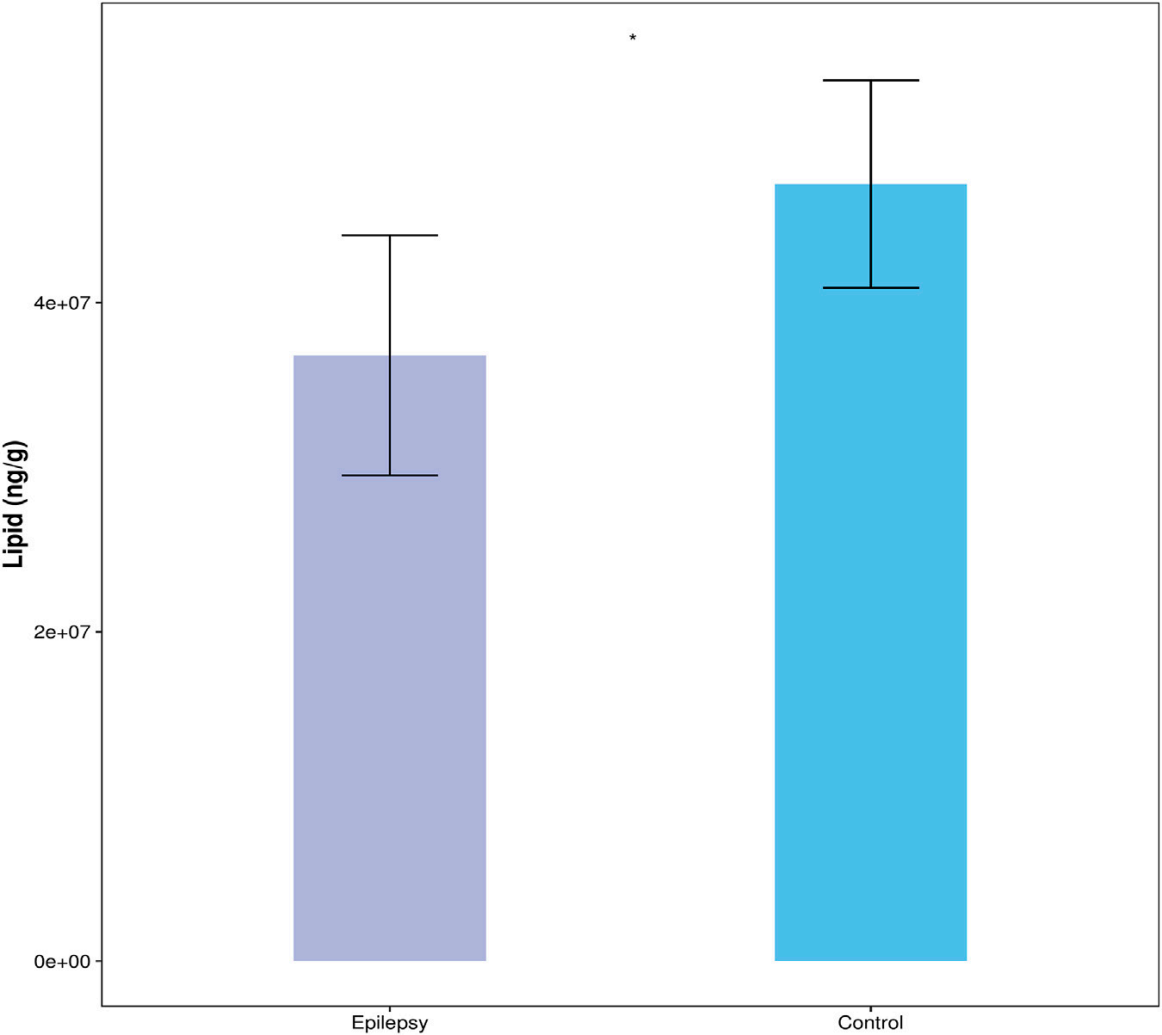
Total lipid molecule content.

The content of all quantified lipid molecules in the same sample is added to obtain the total content of lipid molecules in the sample. Then, the total contents of the blank control group and the epilepsy group can be compared, and the difference of the total lipid molecule content can be visually displayed in the form of a bar chart, as shown in Figure 2. Compared with the control group, the content of lipid molecules in the hippocampus of the TLE group was significantly reduced.

### 3.2 Class analysis

**FIGURE 3:**
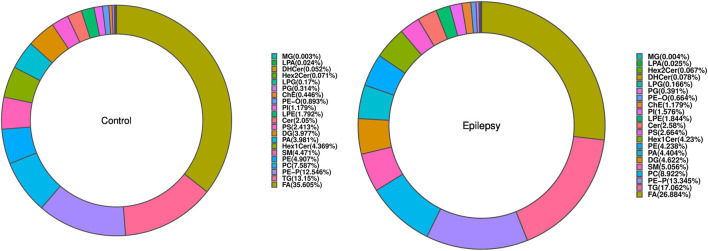
Composition of lipid classes.

The composition of lipid subclasses of the samples in each group is shown in a ring diagram (Figure 3). A circular graph corresponds to a set of samples. The first few lipid subclasses with a higher proportion are the main lipid components of the sample. In Figure 3, compared with that in normal rats, the lipid contents of FA, LPA, PE, and PE-O in the hippocampus of epileptic rats were decreased, and the lipid contents of ChE and DHCer were increased.

### 3.3 Species-level analysis

**FIGURE 4:**
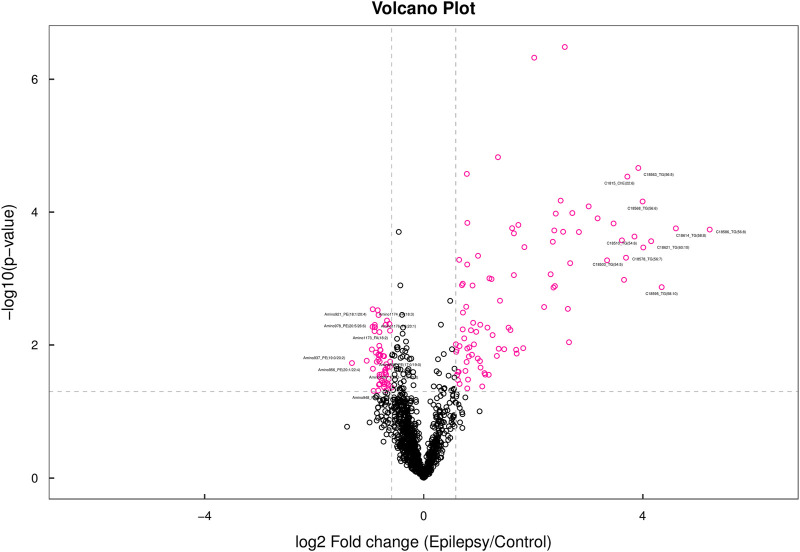
Volcano map. In the volcano map, lipid molecules with a *p*-value less than 0.05 are shown in red, while other metabolites are depicted in black. The lipid molecules highlighted in the figure represent the top 10 metabolites with the most significant upregulated and downregulated fold changes (FC).

When all the detected lipid molecules were analyzed for differences, a total of six lipid subclasses (including 31 species) showed significant differences in the content. The detailed data are shown in Supplementary Figures S1–S6. We display the results in the form of a volcano map (Figure 4). Differential lipid molecules that meet fold change (FC) > 1.5 or FC < 0.67 and *p*-value <0.05 are represented by different colors. The top 10 lipid molecules with upregulated FC and the top 10 with downregulated FC were selected for labeling.

**FIGURE 5:**
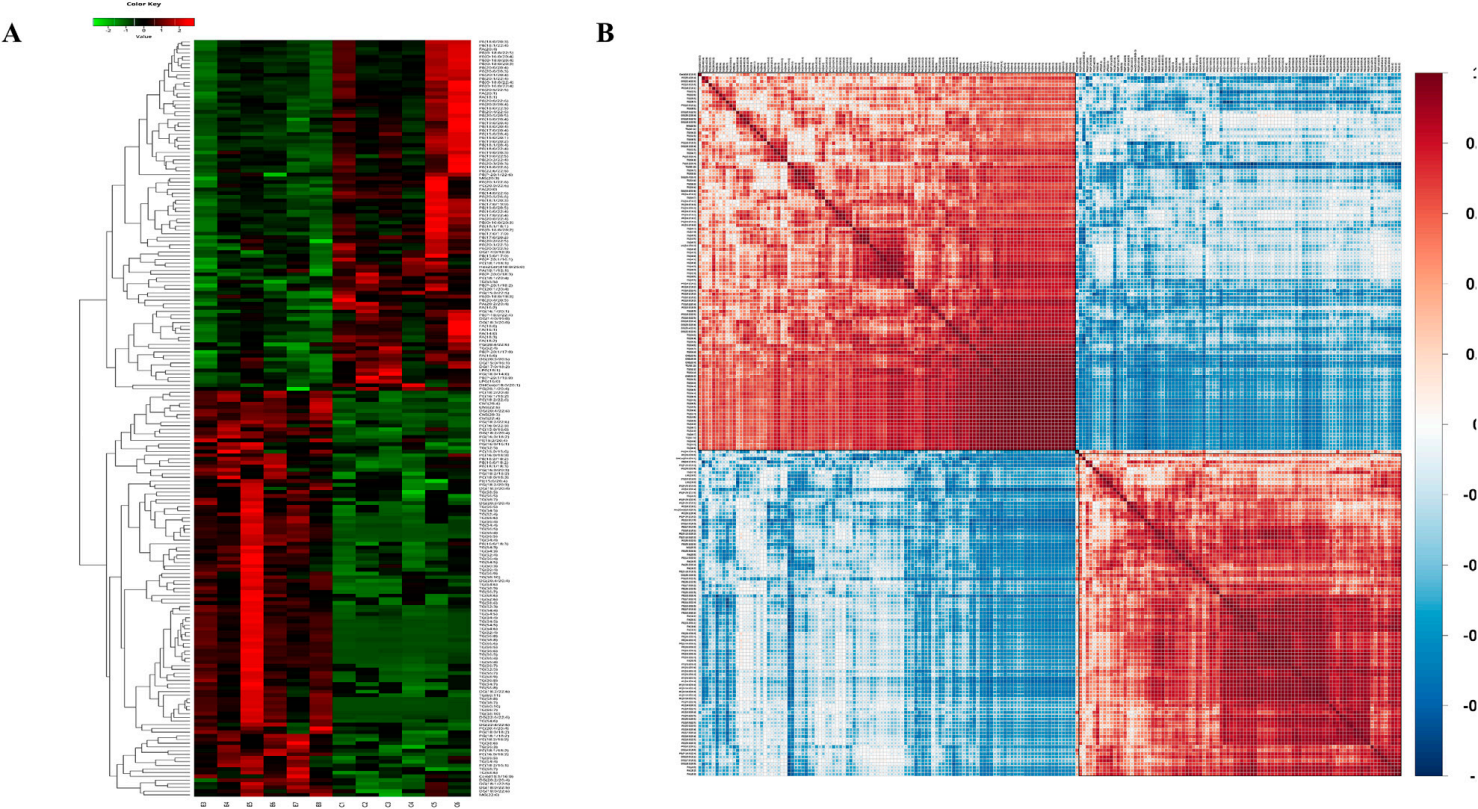
**(A)** Clustering heat map; **(B)** Correlation clustering heat map.

We then aimed to elucidate the relationship between the samples and differences in the expression patterns of lipids in different samples (VIP>1). The *p-*values <0.05 were used for hierarchical clustering (Figure 5A). In the clustering heat map, the significantly differentially expressed lipid molecules in the epilepsy group were highly expressed when compared with that in the control group in the red region and less expressed in the blue regions. The associations between the lipids with significant difference were analyzed using the correlation analysis method. The results of correlation analysis are visualized as a correlation clustering heat map (Figure 5B). Red indicates a positive correlation, and blue indicates a negative correlation. Lipids with correlational expression may jointly participate in biological processes; in addition, a positive correlation between lipids may also indicate that they originate from the same synthetic pathway, and a negative correlation may indicate that they are broken down for the synthesis of other lipids.

### 3.4 Lipid chain length analysis

On taking the lipid molecule DG (16:0/16:0/0:0) as an example, the chain length is the sum of the C atoms of the fatty acid chain possessed by the lipid molecule, that is, the length is 32 carbon atoms. In addition to the lipid content and lipid function, lipid chain length is also an influential factor that cannot be ignored: the chain length affects the thickness of the cell membrane, and then it affects the fluidity of the cell membrane and the activity and function of the related lipid transporters and target proteins. According to the statistical differences in the number of carbon atoms in different subclasses, we found that there were 10 classes of lipids with different carbon atom content, namely, Cer (1), ChE (2), DG (2), FA (4), LPG (1), PC (1), PE (8), PG (1), PI (1), and TG (3).

### 3.5 Lipid saturation analysis

On taking the lipid molecule TG (18:1/18:1/18:1) as an example, chain saturation is the sum of the number of double bonds of the fatty acid chain possessed by the lipid molecule, that is, the unsaturation is 3. In addition to the above lipid content and chain length, lipid saturation is also an important factor affecting lipid function. According to the statistical difference of chain saturation of different subclasses, we found that there were 13 types of lipids with different carbon atom contents, namely, ChE (3), DG (4), FA (5), LPA (1), LPG (1), MG (2), PA (2), PC (2), PE(7), PE-O (2), PE-P (1), PG (1), and TG (7).

### 3.6 Lipid KEGG analysis

**FIGURE 6:**
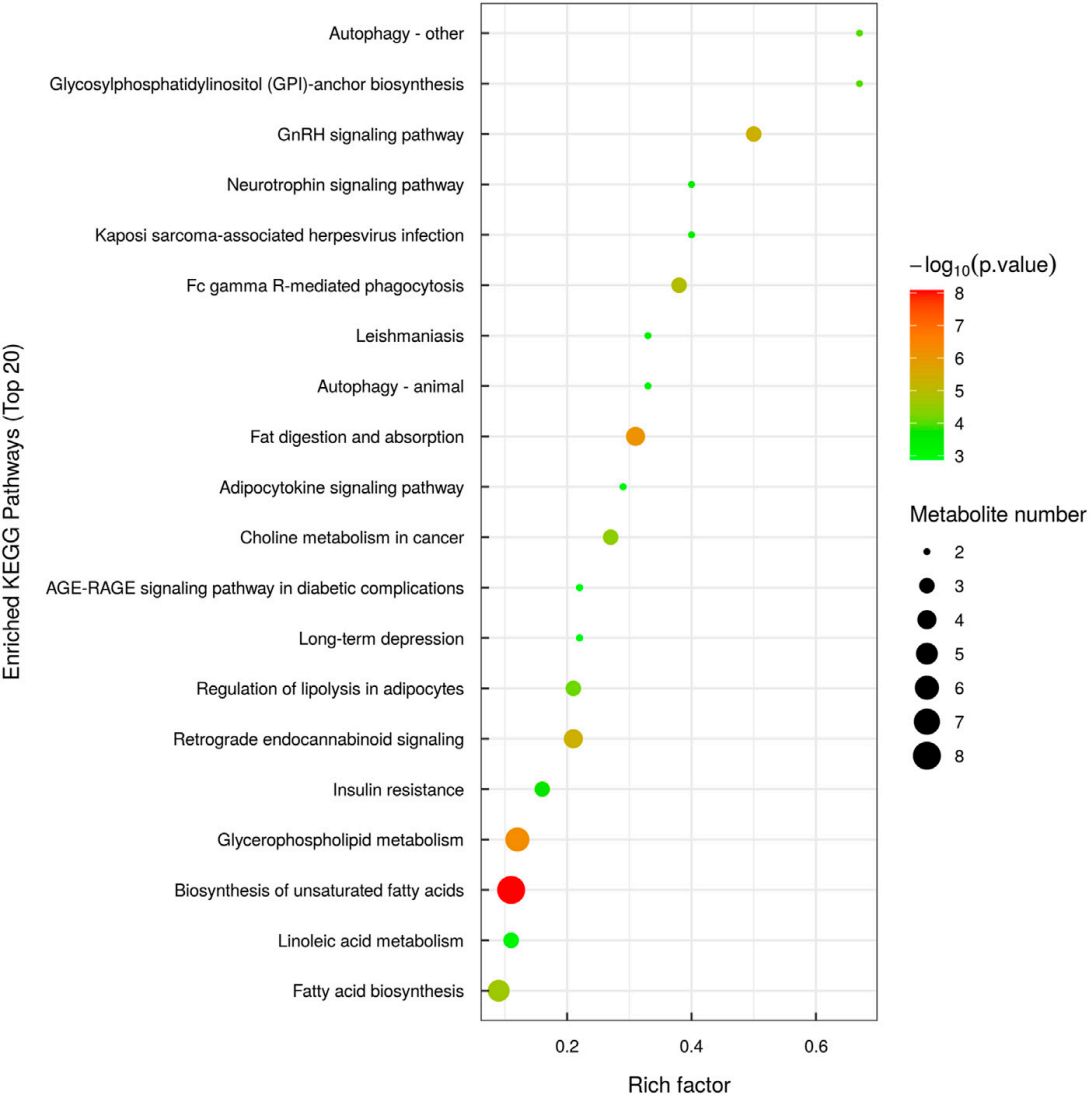
Bubble diagram illustrating KEGG pathway enrichment.

KEGG pathway enrichment analysis is based on the KEGG pathway as a unit and the metabolic pathways involved in this species or closely related species as the background. Fisher’s exact test is used to analyze and calculate the significance level of metabolite enrichment in each pathway. The *p*-value was used to identify the metabolic and signal transduction pathways that are significantly affected: the smaller the *p*-value, the more significant the difference of the metabolic pathway. The results of the metabolic pathway enrichment analysis are shown in the form of a bubble diagram, as shown in Figure 6.

### 3.7 Analysis of the overall changes of the KEGG metabolic pathway

**FIGURE 7:**
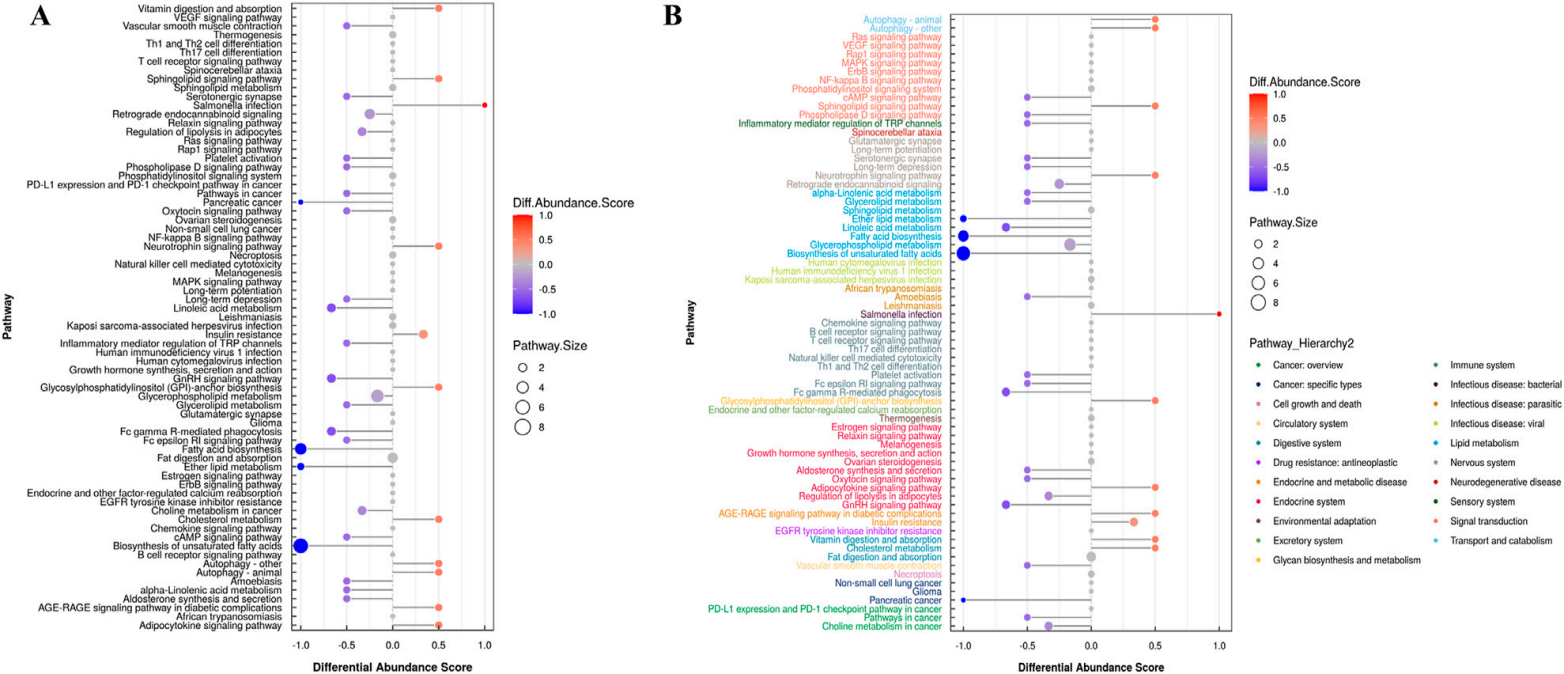
**(A)** Graph depicting differential abundance scores across various metabolic pathways. **(B)** Differential abundance scores for all metabolic pathways, categorized by Pathway_Hierarchy2. (The larger the dot, the greater the number of metabolites).

The differential abundance score is a pathway-based method for analyzing metabolic changes. The differential abundance score can capture the average and overall changes of all metabolites in a pathway. Using the example comparison group as an example, the differential abundance scores for all the differential metabolic pathways are shown (Figure 7A). All the differential metabolic pathways are classified according to their upper level Pathway_Hierarchy2, and then rehierarchy2 are displayed, and the results are shown in Figure 7B.

In Figure 7B, the size of the dot at the end of the line segment indicates the number of metabolites in the pathway, and larger dots indicate more metabolites. Darker red indicates upregulated expression of the pathway, and darker blue indicates downregulated expression. We found that the largest number of metabolites was involved in the biosynthesis of unsaturated fatty acids in the hippocampus of epileptic rats, and the overall expression of this pathway tended to be upregulated. The overall expression of the pathway tended to be upregulated, and the autophagy pathway involved the most metabolites.

## 4 Discussion

In this study, the targeted lipidomics method based on UHPLC–MS/MS technology was used to perform absolute quantitative analysis of the lipidomics of the samples. The results of the quality control evaluation show that the instrument analysis system of this experiment is stable and reliable. Data analysis at multiple levels, such as lipid content, chain length, saturation, and KEGG analysis, systematically, comprehensively, and deeply revealed the differences between the groups, which could be further revealed by combining the lipid function molecular mechanisms of related biological processes and phenotypes.

Previous clinical studies have found that abnormal metabolism of TG, cholesterol, sphingolipid, phospholipid, and bile acids is a key factor in the progression of epilepsy (Wei et al., 2022; Walimbe et al., 2024; Guo et al., 2023). The differential lipid results in the above studies may differ from ours due to several reasons. First, the samples of these studies were derived from the serum or plasma of patients, and our study was mainly based on the hippocampus of epileptic rats. Second, the patients in these studies included the elderly and children, and our study was based on adult male rats. Finally, these patients were already taking antiepileptic drugs, some of which may affect plasma or serum lipid levels (Okada et al., 2020). Using mass spectrometry imaging of 39 freshly frozen human hippocampal surgical specimens, Ajith et al. found reduced expression of various important lipids in TLE hippocampus, especially PC and PE (Ajith et al., 2021), which is consistent with some of our findings.

Our previous study identified damage to the hippocampal CA3 region of epileptic rats 24 h after seizure, so we chose 24 h after seizure as the time point for sampling (Zhao et al., 2023). These differentially expressed lipids may not return to normal levels in the short term. Studies have shown that after epileptic seizures, lipid metabolism in the brain may undergo reprogramming, leading to lipid accumulation in astrocytes, which may persist for a certain period. For example, in a study conducted by Chen et al. in 2023, it was found that apolipoprotein E-mediated lipid transfer occurs from hyperactive neurons to astrocytes in the brains of epilepsy patients, resulting in lipid metabolism reprogramming and the formation of lipid-accumulated reactive astrocytes (LARA). These astrocytes exacerbate abnormal discharges in neighboring neurons, aggravate seizure symptoms in mice, and contribute to disease progression (Chen et al., 2023). In addition, neuroinflammation is often associated with seizures, and certain inflammatory factors may continue to alter lipid metabolism. TNF-α is a typical pro-inflammatory factor that promotes the breakdown of triacylglycerol by activating hormone-sensitive lipase (HSL) in adipose tissue (Sharma and Puri, 2016). IL-6 reduces lipid synthesis in the liver by down-regulating the expression of fat synthase enzymes (e.g., fatty acid synthases) and genes involved in lipid synthesis (e.g., *SREBP-1c*). During acute inflammatory responses, elevated IL-6 may promote changes in fat metabolism, which subsequently affects lipid accumulation and metabolic function in the liver (Dumas et al., 2019; Farhadi et al., 2021; Li et al., 2015). TNF-α and IL-6 induce reprogramming of lipid metabolism in the brain, leading to the accumulation of lipids in astrocytes, further exacerbating inflammation (Chen et al., 2023).

Abnormal lipid metabolism may be present for extended periods after a seizure. Chen et al. found that LARA may reduce glutamate reuptake in the brains of patients with temporal lobe epilepsy by upregulating the expression of the adenosine 2A receptor, thus increasing the excitability of neurons. Repeated seizures may further lead to abnormal lipid metabolism (Chen et al., 2023). Research on the regulation of lipid metabolism by microglia in epilepsy is relatively limited. Microglia and astrocytes secrete associated inflammatory factors (TNF-α, IL-6, etc.) after seizures. Moreover, the release of TNF-α promotes the activation of astrocytes, which in turn affects their ability to process lipids. We found that TNF-α not only promoted glutaminase expression in astrocytes but also increased the release of extracellular vesicles containing lipids and signaling molecules, which may play a role in neuroinflammation and intercellular communication (Wang et al., 2017). For patients with epilepsy, long-term monotherapy with antiepileptic drugs can affect blood lipid levels. A study showed that carbamazepine and phenytoin sodium had a significant effect on blood lipid levels, especially total cholesterol and low-density lipoprotein cholesterol (LDL-C) levels. However, the effects of valproic acid and lamotrigine monotherapy on total cholesterol and LDL-C levels were not significant (Chuang et al., 2012).

Our study found that ChE and DHCer expression increased, while that of FA, LPA, PE, and PE-O decreased in the hippocampus of epileptic rats. These lipids may be related to neuronal injury and cell membrane repair. Epileptic seizures can trigger inflammation in the brain, and activated microglia and astrocytes secrete cholesterol esters through cholesterol metabolism pathways to respond to inflammation and repair cell membranes. Epilepsy causes neuronal membrane damage; ChE and DHCer are major components of the membrane and neuronal membrane stability, respectively. Increases in both may be related to the remodeling of synapses and membranes and neuronal repair. During seizures, energy requirements increase. Seizures may lead to impaired mitochondrial function, affecting the normal metabolism of fatty acids, which in turn leads to reduced energy production. In addition, during seizures, neuronal activity is abnormally heightened, leading to sharp increases in energy requirements. To meet this demand, cells may accelerate the oxidation of FA to provide more ATP, leading to decreased fatty acid levels. Studies have shown that in some neurological diseases, energy metabolism may shift from glucose metabolism to FA oxidation. For example, in the hippocampus that lacks sortilin-related receptor 1, energy metabolism shifts from glucose to fatty acid oxidation, which subsequently leads to elevated ROS levels, potentially triggering further cell damage (Wang et al., 2024). LPA plays an important role in regulating neuronal activity and inflammatory responses. Epilepsy may inhibit its production and alter the balance of phospholipid metabolism. Epilepsy may lead to membrane destruction, and PE and PE-O are consumed in large quantities to repair damaged nerve membranes, which affects the activity of metabolic enzymes related to PE synthesis (such as ethanolamine phosphotransferase) and reducing PE production (Vance, 2008).

According to the KEGG pathway analysis, lipid metabolism in the hippocampus of epileptic rats primarily occurred through the autophagy, gonadotropin-releasing hormone (GnRH), and glycosylphosphatidylinositol (GPI) pathways. Autophagy begins with the formation of an isolated membrane structure called a phagocytic mass, which expands by acquiring lipids and eventually closes to form an autophagosome. Various organelles, including endoplasmic reticulum, mitochondria, plasma membrane, and Golgi apparatus, have been implicated in providing membrane lipid sources for autophagosomes (Shibutani and Yoshimori, 2014; Gómez-Sánchez et al., 2021). A recent study showed that phagophore expansion requires local fatty acid channeling into phospholipid synthesis during the autophagic process (Schütter et al., 2020). In mammalian cells, the patatin-like phospholipase domain-containing protein 5 (PNPLA5) TAG lipase and several enzymes involved in phospholipid biosynthesis are positive regulators of autophagy, implying that neutral lipid stores are the key lipid source for autophagic membrane formation (Dupont et al., 2014). A transmembrane protein TMEM41B may also be involved in lipid exchange between lipid droplets (LDs) and autophagosomes (Moretti et al., 2018). This study also showed that LDs are not required as a membrane lipid source for autophagosome biogenesis but function in autophagy regulation by buffering fatty acid-induced lipotoxic stress to maintain ER lipid homeostasis, intact autophagy, and cell viability (Shpilka et al., 2015). A recent study showed that the synthesis of autophagic membranes requires an activation of CTP:phosphocholine cytidylyltransferase (CCT) on autophagy-derived LDs (Ogasawara et al., 2020). Overall, available data indicate that further research is required to define the exact role of fatty acid metabolism in autophagy and cellular homeostasis and the potential function of LDs in autophagy.

GnRH is a decapeptide hormone secreted by the hypothalamus. Its sequence is highly conserved in mammals, and its main physiological function is to promote the synthesis and secretion of luteinizing hormone (LH) and follicle-stimulating hormone (FSH) by activating the GnRH receptor in pituitary cells, which in turn act on reproductive organs such as ovaries or testes to regulate gametogenesis and sex hormone secretion, finally forming the hypothalamus–adenopituitary–gonad axis (Wu et al., 2021; Gründker and Emons, 2021). Women with epilepsy using antiepileptic drug VPA often suffer from reproductive endocrine disorders, menstrual disorders, and polycystic ovaries (Røste et al., 2005; Svalheim et al., 2003). Valproic acid exerts anticonvulsive effects via the gamma amino butyric acid (GABA) neurotransmitter system, which also acts as a neurochemical regulator of GnRH neurons and suggests the possibility of valproic acid-mediated interruption in gonadotropin-releasing hormone pulse generator in the hypothalamus. Lakhanpal et al. observed GnRH and GABA expression, neural cell adhesion molecule, and ovarian histological changes in female rats, suggesting that VPA treatment disrupts the hypothalamic–pituitary–gonadal axis (HPG) at the level of the hypothalamic GnRH pulse generator (Lakhanpal and Kaur, 2007). The GnRH receptor is expressed in fat cells. As fat cells mature, the GnRH receptor expression increases gradually. Multiple studies have found significant differences in the prevalence of obesity between women with epilepsy and healthy controls (Li et al., 2024; El-Khayat et al., 2004; Ayyagari et al., 2012). Due to the limited number of studies on GnRH and epilepsy, the mechanism of GnRH’s involvement in epilepsy needs more research.

GPI is a glycolipid, which anchors 150 or more types of proteins to the cell surface (Kinoshita, 2020). Phosphatidylinositol glycan biosynthesis class A protein (PIGA) catalyzes the very first step of GPI anchor biosynthesis. Patients carrying a mutation of the *PIGA* gene usually suffer from inherited glycosylphosphatidylinositol deficiency (IGD) with intractable epilepsy and intellectual developmental disorder. Developmental and epileptic encephalopathies include a range of severe epilepsies in which intractable seizures are accompanied by the impairment of motor and cognitive functions (Scheffer and Liao, 2020). Early infantile epileptic encephalopathy‐55, a severe form of epilepsy with frequent tonic seizures or spasms beginning in infancy and developmental delay or regression, has been linked to several genes that are important for GPI biosynthesis and attachment. For example, *PIGB*, *PIGQ*, *PIGP*, and *PGAP1* have all been linked to novel autosomal recessive IGDs (Murakami et al., 2019; Martin et al., 2014; Johnstone et al., 2017). Kandasamy et al. generated three mouse models with PIGA deficits, specifically in telencephalon excitatory neurons (Ex-M-cko), inhibitory neurons (In-M-cko), or thalamic neurons (Th-H-cko). Both Ex-M-cko and In-M-cko mice showed impaired long-term fear memory and were more susceptible to kainic acid-induced seizures (Kandasamy et al., 2021). It has been commonly assumed that an imbalance between the excitatory and inhibitory synaptic transmission in the brain initiates seizure activity. Future studies will delve into the morphological, molecular, and physiological changes of excitatory and inhibitory neurons in these mutants to better understand the disease.

This research has some limitations. First, the study performed lipidomics analyses only at a single point in time (24 h after KA induction), which limits our understanding of changes in lipid metabolism over time. Future efforts to observe changes at multiple time points will better reveal the dynamics of lipidomics after seizures, including differences in short- and long-term effects. Second, using only normal rats as a control group did not adequately control for the effects of the injection procedure itself. The introduction of a sham operation group as a control group can better rule out inflammation and metabolic reactions that may be triggered during the injection process and thus more accurately assess the specific effects of KA-induced epilepsy on lipid metabolism. Finally, this study only focused on the changes of lipid metabolism in hippocampal neurons, and attention should be paid to the metabolic changes of astrocytes and microglia and their effects on lipid metabolism in neurons in future studies.

## 5 Conclusion

In this project, 21 subclasses of lipids were detected using the UPLC–MS/MS detection platform. Different lipid changes in focal epilepsy hippocampus tissues, such as increased ChE and DHCer levels and a decrease in FA, LPA, PE, and PE-O, may be related to an increase in the components constituting the membrane structure required for cell proliferation and the rearrangement of events in energy metabolism; this aspect warrants further in-depth verification.

## Data Availability

The original contributions presented in the study are publicly available. This data can be found here: https://doi.org/10.6084/m9.figshare.28112816.
