# Supplementary figures and images for: Targeted lipidomics analysis of possible molecular mechanisms of lipid changes in temporal lobe epilepsy models
Source: Front Pharmacol. 2025 Jan 9;15:1531524. doi: 10.3389/fphar.2024.1531524 (PMC11754250; doi:10.3389/fphar.2024.1531524)

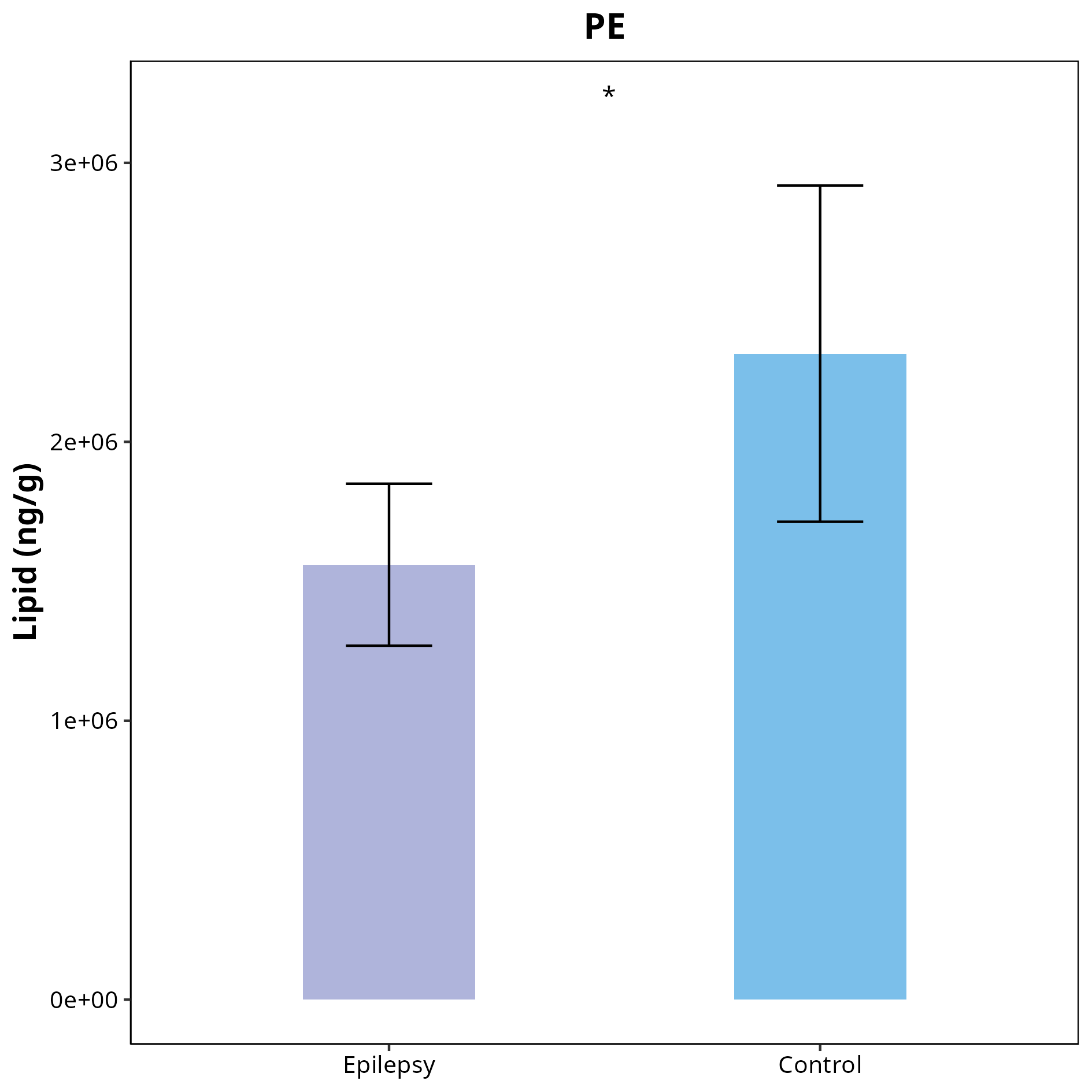

Supplement: Supplementary file 1 [file Image5.PNG]

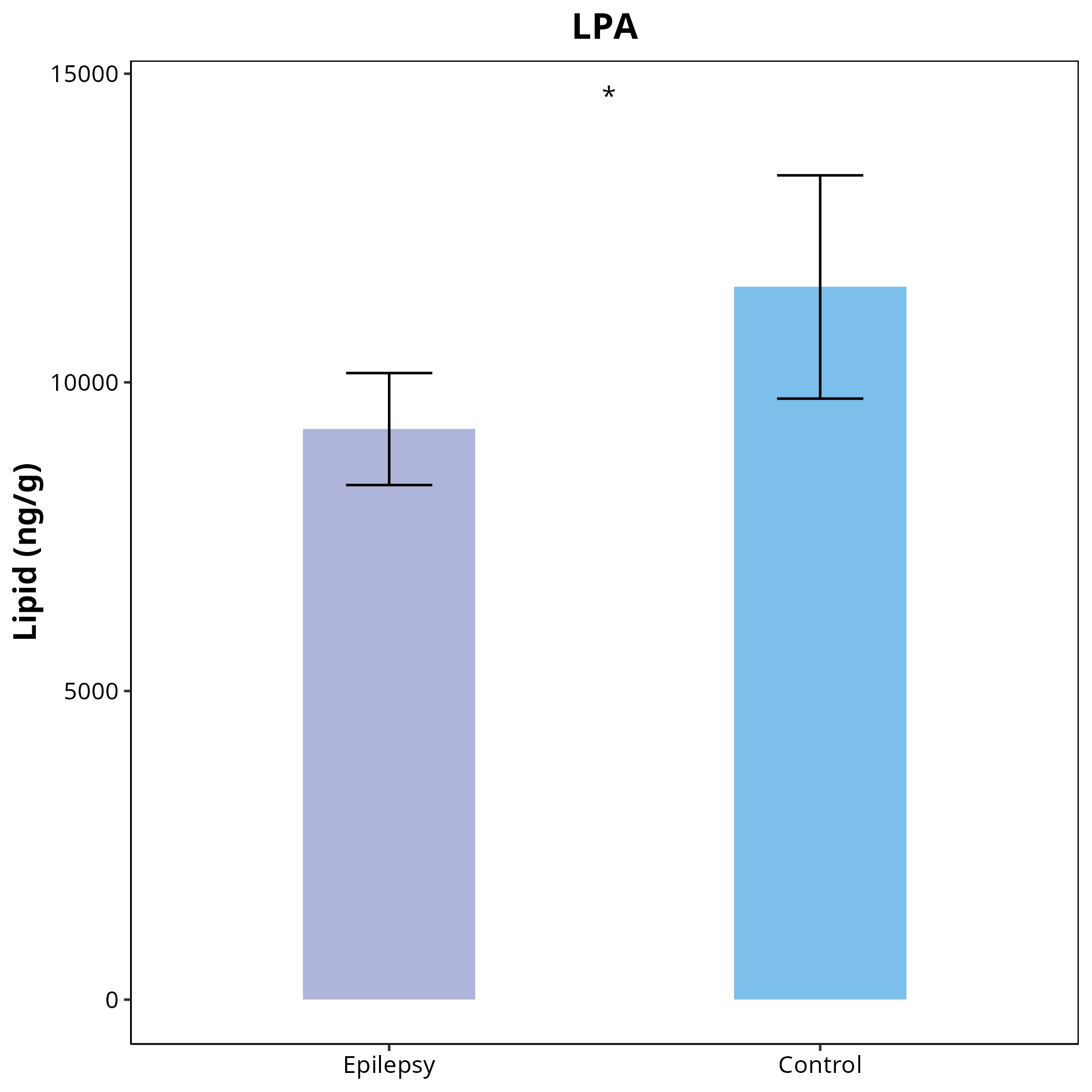

Supplement: Supplementary file 2 [file Image4.PNG]

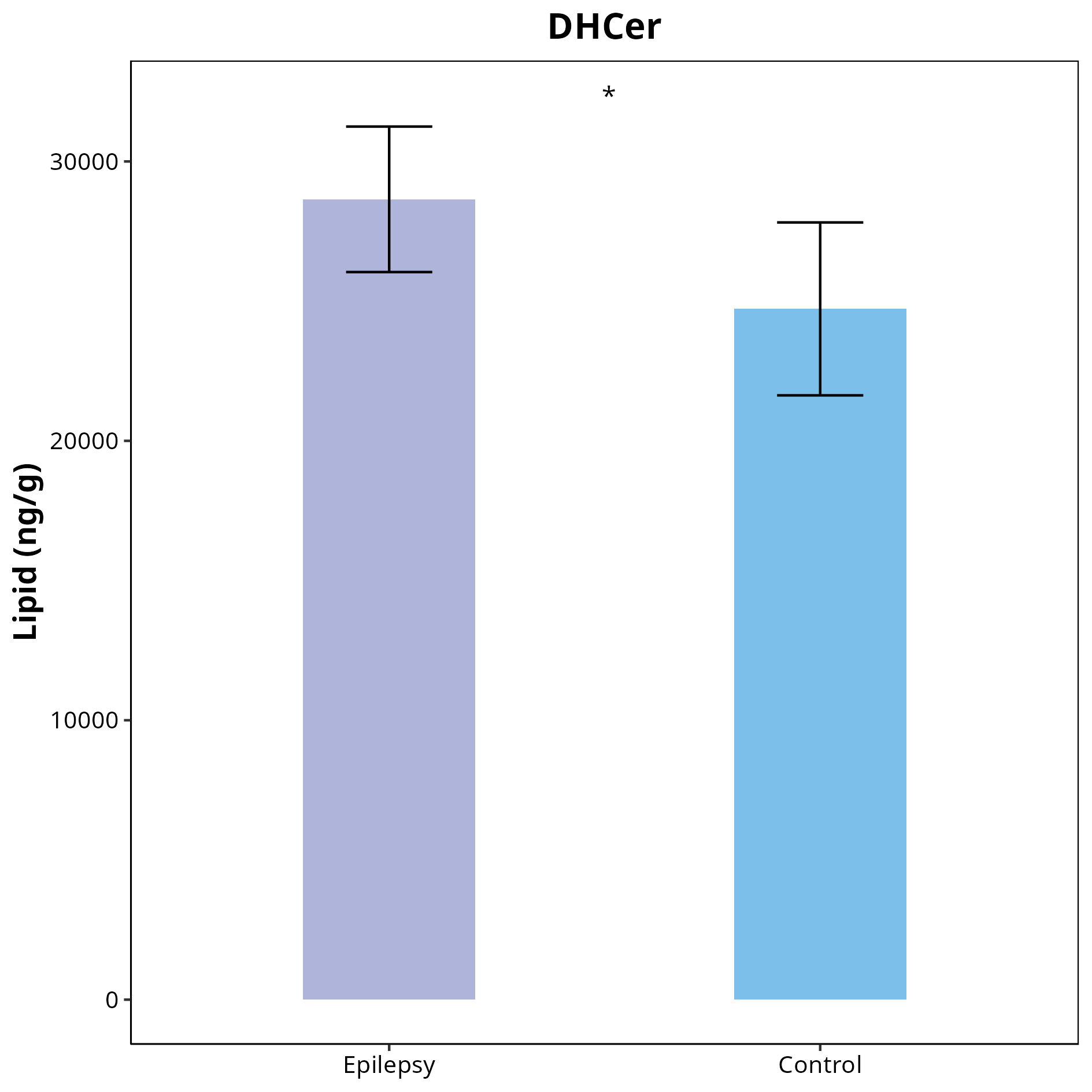

Supplement: Supplementary file 3 [file Image2.PNG]

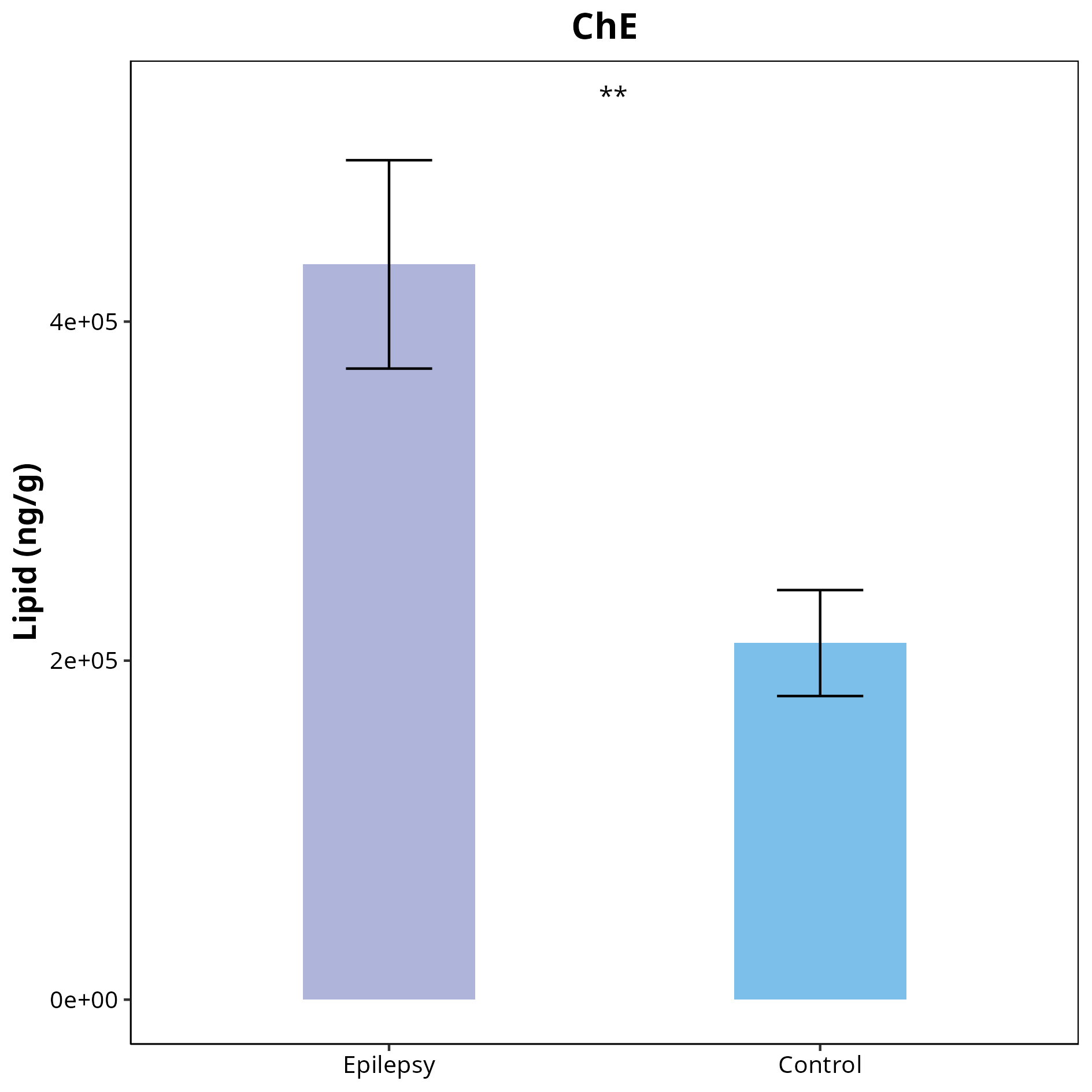

Supplement: Supplementary file 4 [file Image1.PNG]

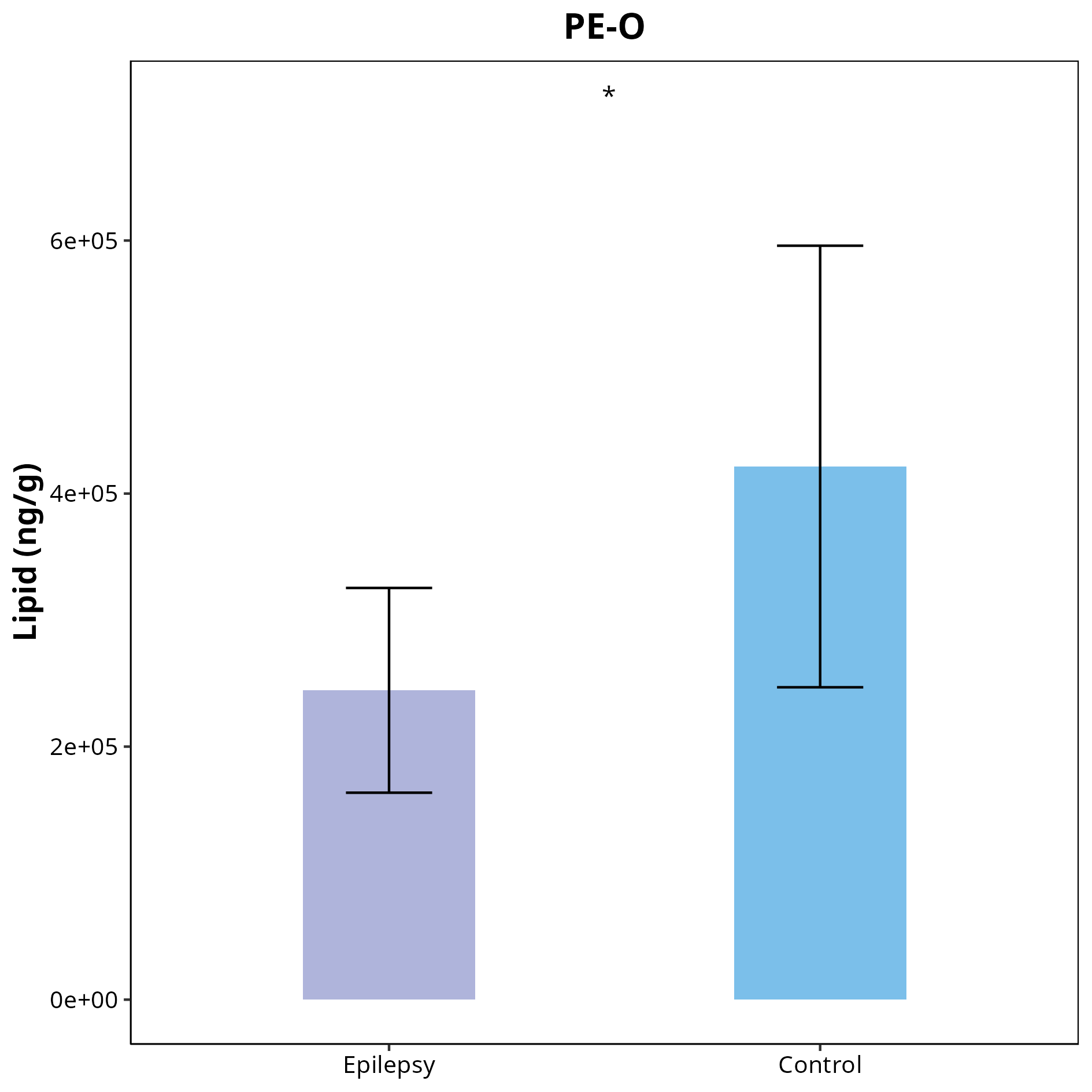

Supplement: Supplementary file 5 [file Image6.PNG]

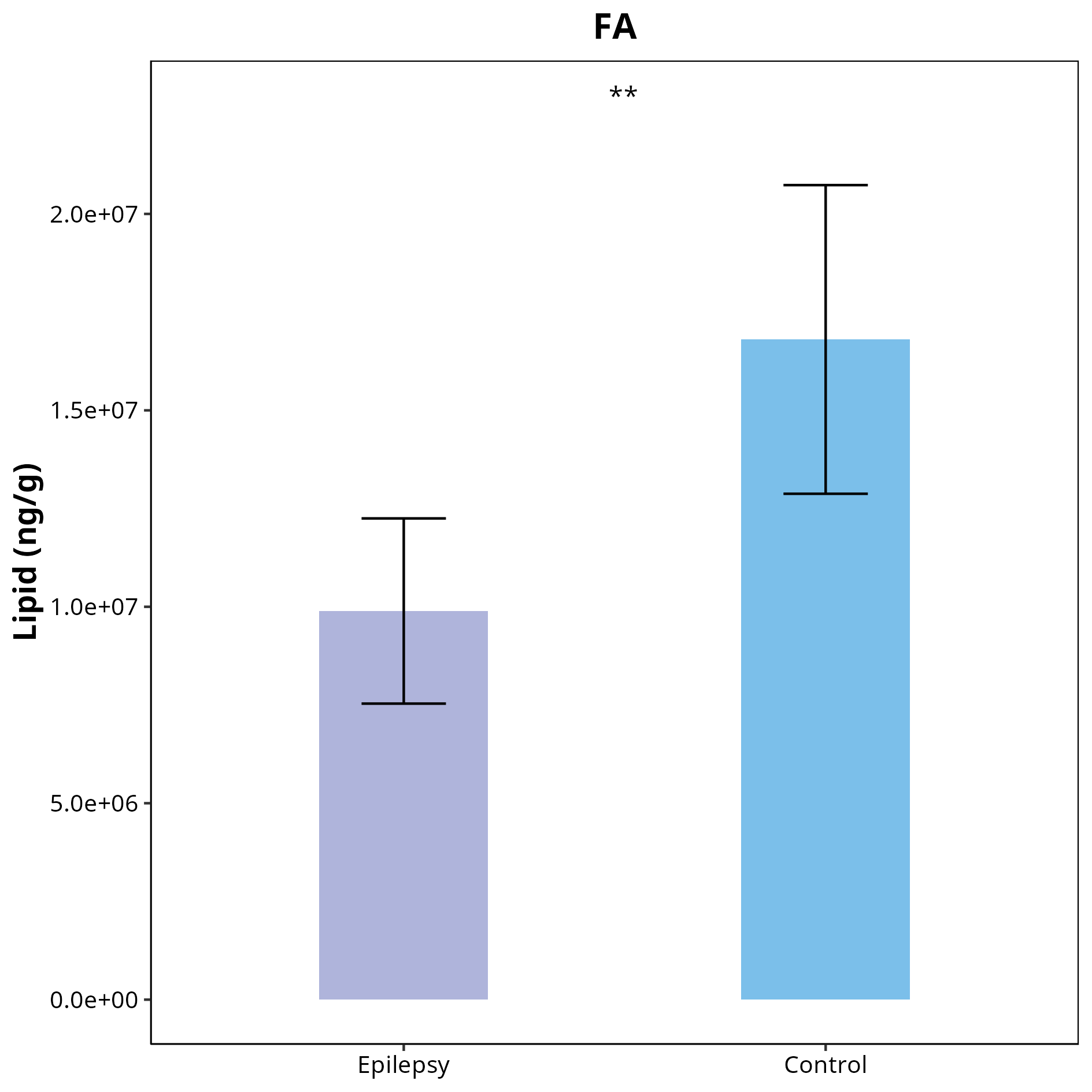

Supplement: Supplementary file 6 [file Image3.PNG]
